# Understanding Vocalization Might Help to Assess Stressful Conditions in Piglets

**DOI:** 10.3390/ani3030923

**Published:** 2013-09-12

**Authors:** Alexandra Ferreira da Silva Cordeiro, Irenilza de Alencar Nääs, Stanley R. M. Oliveira, Fabio Violaro, Andréia C. M. de Almeida, Diego Pereira Neves

**Affiliations:** 1 Agricultural Engineering College, State University of Campinas, Ave. Candido Rondon, 501, Campinas, SP, 13083-875, Brazil; E-Mails: alexandracordeiro6@gmail.com (A.F.S.C.); decom@fee.unicamp.br (F.V.); andreia.almeida@feagri.unicamp.br (A.C.M.A.); diegopneves@gmail.com (D.P.N.); 2 Embrapa Agricultural Informatics, Ave. André Tosello, 209, Campinas, SP, 13083-886, Brazil; E-Mail: stanley.oliveira@embrapa.br

**Keywords:** animal welfare, pig farming, sound signals, classification algorithm

## Abstract

**Simple Summary:**

This research aimed to analyze the possibility of assessing piglets’ welfare using the records of their vocalization. The trial was done in a pig commercial farm, and we recorded the vocal signals from piglets in several stressful exposure situations. Data mining techniques were applied to the processed signals in order to obtain a stress classification using the recorded data. We found that, using the piglets’ vocalization, it was possible to identify the most frequent stressful conditions at the farrowing phase, namely: pain, cold and hunger.

**Abstract:**

Assessing pigs’ welfare is one of the most challenging subjects in intensive pig farming. Animal vocalization analysis is a noninvasive procedure and may be used as a tool for assessing animal welfare status. The objective of this research was to identify stress conditions in piglets reared in farrowing pens through their vocalization. Vocal signals were collected from 40 animals under the following situations: normal (baseline), feeling cold, in pain, and feeling hunger. A unidirectional microphone positioned about 15 cm from the animals’ mouth was used for recording the acoustic signals. The microphone was connected to a digital recorder, where the signals were digitized at the 44,100 Hz frequency. The collected sounds were edited and analyzed. The J48 decision tree algorithm available at the Weka^®^ data mining software was used for stress classification. It was possible to categorize diverse conditions from the piglets’ vocalization during the farrowing phase (pain, cold and hunger), with an accuracy rate of 81.12%. Results indicated that vocalization might be an effective welfare indicator, and it could be applied for assessing distress from pain, cold and hunger in farrowing piglets.

## 1. Introduction

Although it is of great relevance, the assessment of animal welfare status in commercial farming is still a difficult task. The Farm Animal Welfare Council (FAWC) proposed the “Five Freedoms” in order to guarantee the minimal welfare conditions in which the animals must be free from thirst, hunger and malnutrition; free of discomfort, pain, injury and disease; should have the freedom to express normal behavior; and be free from fear and stress. By this approach, it is necessary to develop accurate tools to estimate the animal welfare status and to ensure applicability on field.

Distress related vocalization is of particular interest as an indicator of impaired welfare [1,2], and it is an objective and noninvasive procedure that has been studied to estimate pigs’ welfare [3,4,5,6]. There are other techniques to recognize stress in animals, e.g., a stress response as an adrenaline release can be accompanied by changes in the rates of specific types of vocalization [7] while other studies suggested an increased rate of vocalization in pigs after receiving corticotrophin injections [8]. Subsequently, increased vocalization was observed in piglets from sows treated with cortisol [9]. According to [2], the energy emission parameters, frequency, and duration of calls are particularly suitable for characterizing the type of call. To analyze the data from vocalization records, some methods can be used to assist in the identification of stress situations. These techniques can transform raw data into relevant information and offer support for the decision making process. In particular, data mining techniques have shown promising results in the discovery of knowledge in the field of animal production [10,11,12]. Data mining is a key step in this process by identifying valid, unknown, potentially useful, and understandable patterns [13]. These patterns can be useful to describe unknown structures and/or to predict new situations on animal behavior. 

The objective of this study was to classify different distress circumstances (pain, cold and hunger) from the vocalization of piglets in the farrowing stage by using data mining techniques. 

## 2. Experimental Section

**Figure 1:**
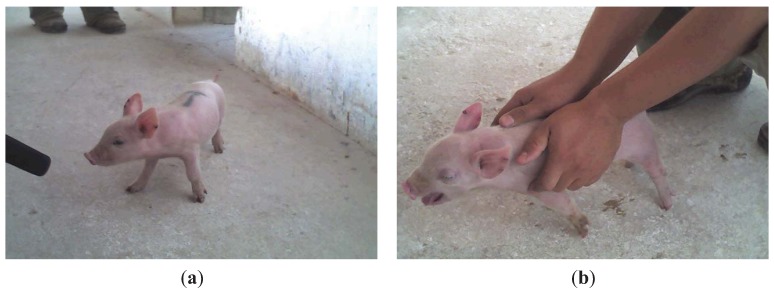
(**a**) Animal isolated in the corridor. (**b**) Assumed pain stress; piglet firmly held by the animal handler.

We recorded individual vocalization from 40 piglets (20 males and 20 females) aged 22 weeks under different stress situations during the farrowing phase. For the sound recording, the animals were randomly selected and individually isolated in a corridor (Figure 1(a)) with limited visual contact with others through a slatted wall. Vocal signals were recorded from the animals under the following situations: normal (baseline, within their thermal comfort zone, pain-free and with access to nursing); followed by the pain distress (consisting of a firmer squeeze than a simple restraint by the animal handler, Figure 1(b)). After the distress by assumed pain, the animals returned to their pens for 2 hours, when normal behavior was observed, and afterwards they were again randomly selected and exposed to the next distress. 

For the thermal stress test, the animals were exposed to a temperature of about 25 °C for 30 min, in which is considered below the thermal comfort zone for piglets during this stage [14]. Under thermal comfort temperature, the animals were apart from each other, indicating the absence of cold. After the cold stress exposure, the piglets became crowded in order to avoid heat loss and keep warm.

**Figure 2:**
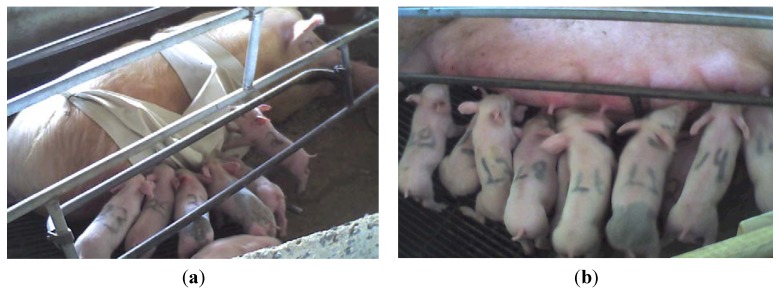
(**a**) Piglets prevented from nursing due to the rubberized fabric. (**b**) Piglets after the nursing constraint.

Similarly to the previous trial, the animals were kept in the pens for 2 hours, prior to the hunger distress. Nursing was constrained from the piglets. The sow’s body was covered with a rubberized fabric tied at the ends with strings, covering the teats (Figure 2(a)); thus, preventing the piglets from nursing. The vocal signal was recorded 30 minutes after the piglets had their last nursing bout. Although this might also indicate frustration for not reaching the sows’ teats, it was assumed they were hungry as they approached the sow to nurse immediately after the removal of the rubberized fabric (Figure 2(b)). 

A Yoga^®^ unidirectional microphone positioned about 15 cm from the animals’ mouth was used for recording the acoustic signals. The microphone was connected to a Marantz^®^ PMD 660 digital recorder, where the signals were digitized at the 44,100 Hz frequency. The recorded sounds were edited and analyzed by using the Praat^®^ software. Each signal was divided into three samples, and for each sample had 20 attributes extracted related to sound, sex identification, and stress situation (Table 1). To determine the stress conditions, the data were processed using Weka^®^ (3.5) software [15], applying the C4.5 decision tree algorithm, (known as J48 in the Weka^®^ environment), considering cross-validation samples with 10% (10-fold cross-validation), e.g., the initial data were randomly partitioned into 10 mutually exclusive subsets or folds, each of approximately equal size. Training and testing were performed 10 times. For classification, the accurate estimation is the overall number of correct classifications from the 10 iterations, divided by the total number of instances in the initial data. In general, stratified 10-fold cross-validation is recommended for estimating accuracy due to its relatively low bias and variance [16].

This field trial was approved by the Ethics Committee, Unicamp, n. 2224-1/2011.

**Table 1:** Attributes used for the classification of stress in pigs.

Attribute	Unit	Description
Sex	-	Male or Female
Distress	-	Normal (baseline), pain, cold and hunger
Signal energy	Pa²*s	Energy emitted in the sound wave
Signal duration	S	Duration of the sound wave
Maximum range	Pa	Maximum range of the sound wave
Minimum range	Pa	Minimum range of the sound wave
Intensity	dB	Sound wave intensity
Pitch frequency	Hz	Determines the pitch level
First formant	Hz	First formant frequency
Second formant	Hz	Second formant frequency
Third formant	Hz	Third formant frequency
Fourth formant	Hz	Fourth formant frequency
Range	Pa	Difference of maximum and minimum ranges
Sum of the formants	Hz	Sum of the 4 formants frequencies
Average formants	Hz	Average of the 4 formants frequencies
F3-F4	Hz	Difference between the 4th and 3rd formants frequencies
F4-F2	Hz	Difference between the 4th and 2nd formants frequencies
F4-F1	Hz	Difference between the 4th and 1st formants frequencies
F3-F2	Hz	Difference between the 3rd and 2nd formants frequencies
F3-F1	Hz	Difference between the 3rd and 1st formants frequencies
F2-F1	Hertz	Difference between the 2nd and 1st formants frequencies
Sum of the differences	Hertz	Sum of the differences between the formants
Average of the differences	Hertz	Average of the differences between the formants

## 3. Results and Discussion

**Figure 3:**
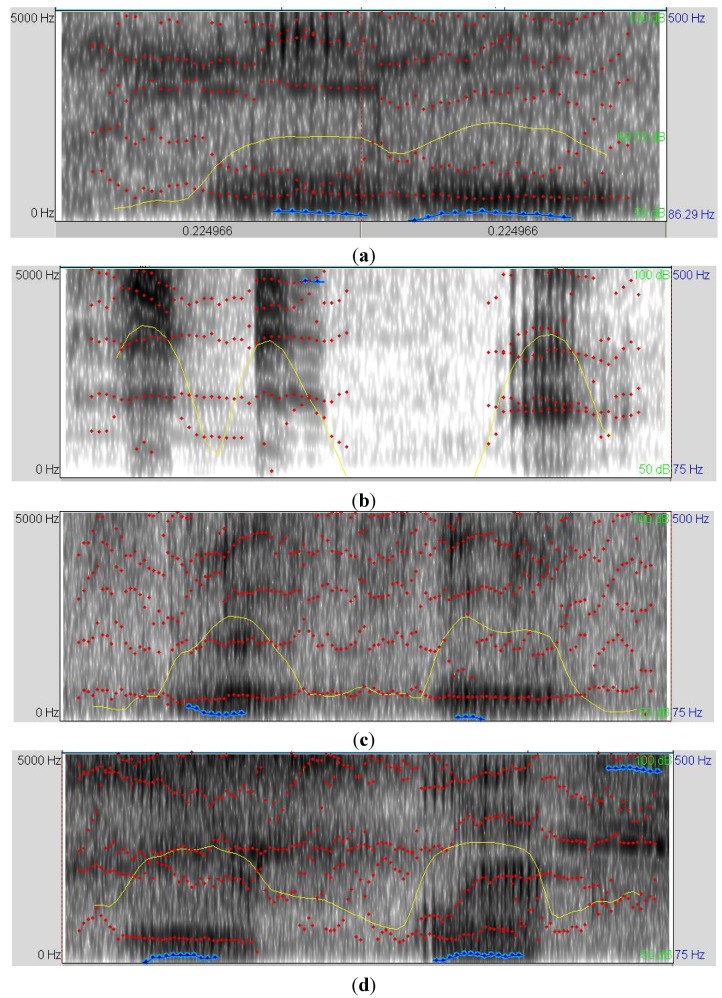
(**a**) Vocalization’s sonogram of piglets under normal welfare (baseline); (**b**) vocalization’s sonogram of piglets being squeezed (assumed to produce pain); (**c**) vocalization's sonogram of piglets feeling cold; (**d**) vocalization's sonogram piglets feeling hunger. The formants (dotted red line), the sound intensity (yellow line), and the frequency of pitch (in blue line) are shown in each sonogram.

Figure 3 shows the vocalizations’ sonogram of piglets in (a) normal welfare status (baseline); (b) in assumed pain; (c) feeling cold, and (d) in hunger distress. The curve of sound intensity showed different profiles for the four studied conditions. Differences in the frequency of pitch were also observed; however, the investigation of the formants differences was more complex. The sonogram represents the initial examination of the data, requiring the extraction of numerical data from these parameters to determine the stress conditions. 

It was possible to classify the four distress conditions from the recorded vocalization, by using the C4.5 algorithm from the Weka^®^ software. In a previous study [10], an algorithm was developed based on Artificial Neural Networks to classify the vocalization of piglets during the farrowing stage, in situations of dispute over the sow’s teats and of eventual risk. 

Table 2 presents the hit rate (%), the Kappa statistic, and the number of rules generated by the J48 algorithm, considering the different pruning levels (minimum number of objects per leaf). The number of objects per leave corresponds to the minimum number of examples of the test set used to evaluate the generated rules (tree leaves). It is observed that in all situations, the Kappa statistic ranged from median (0.4 to 0.75) to excellent (>0.75) [17]. The Kappa statistic procedures is used to measure the reliability between predicted and observed categorizations of a dataset, while correcting for an agreement that occurs by chance. It ranges from 0 to 1, where 0 indicates no confidence and 1 the maximum confidence of the classifier. In particular, the accuracy rate (81.69) yielded the largest Kappa statistic (0.75) for a minimum number of objects per leaf equal to 9. However, this scenario generated the largest number of rules, providing a more complex decision tree. Therefore, for the purpose in question, it is more interesting to increase both the accuracy rate and the Kappa statistic than to get a more simplified decision tree.

**Table 2:** Accuracy rate, Kappa statistic, and the number of rules for the different numbers of objects per leaf.

Minimum number of objects per sheet	Accuracy (%)	Kappa statistic	Number of rules
3	79.72	0.73	7
6	78.54	0.71	15
9	81.69	0.75	12
12	80.17	0.73	9
15	77.95	0.71	10
18	76.97	0.69	6
21	75.59	0.67	6
24	77.75	0.70	6
27	77.75	0.70	5
30	77.95	0.71	5

**Figure 4:**
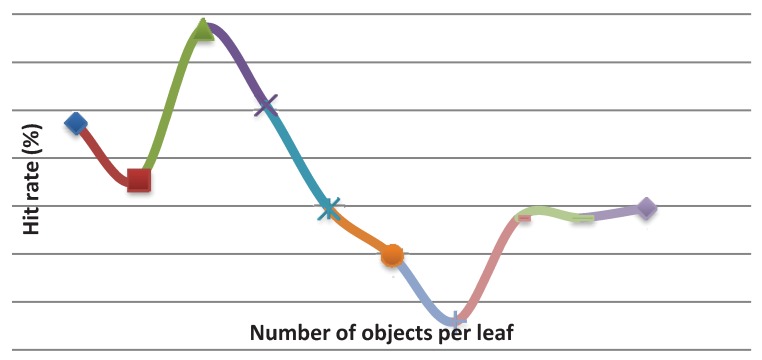
Hit rates (%) *vs.* minimum number of objects per leaf.

**Figure 5:**
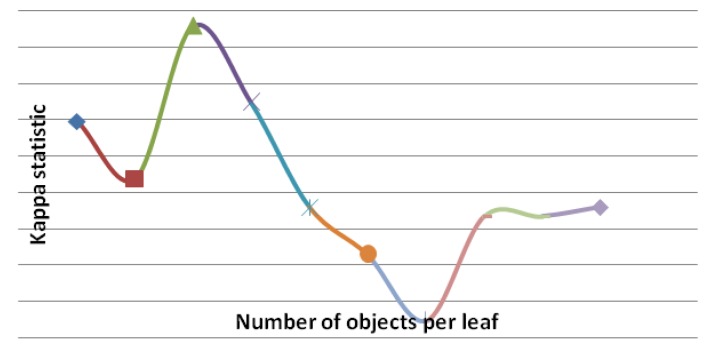
Kappa statistics (%) *vs.* minimum number of objects per leaf.

**Figure 6:**
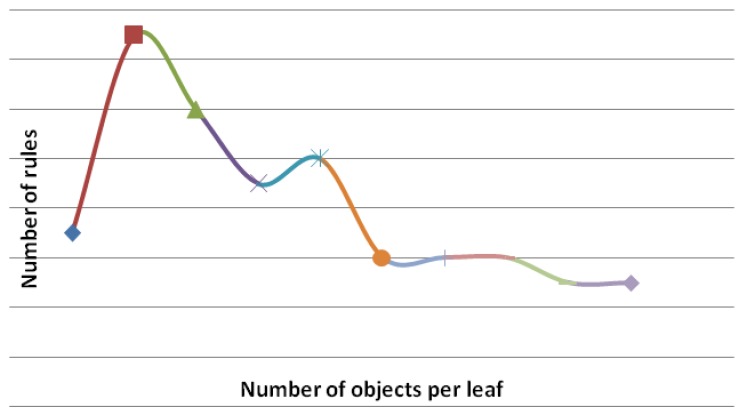
Number of rules (%) *vs.* minimum number of objects per leaf.

An increase of the accuracy rate (%) occurred with the increase of the minimum number of objects per leaf up to the maximum value (82%). Then, the accuracy rate (%) tended to decrease (Figure 4). The same behavior was observed for the Kappa statistic values (Figure 5), and for the number of rules (Figure 6). The results indicated that, for this dataset, the best model (decision tree) was the one whose minimum number of objects per leaf is equal to nine.

**Figure 7:**
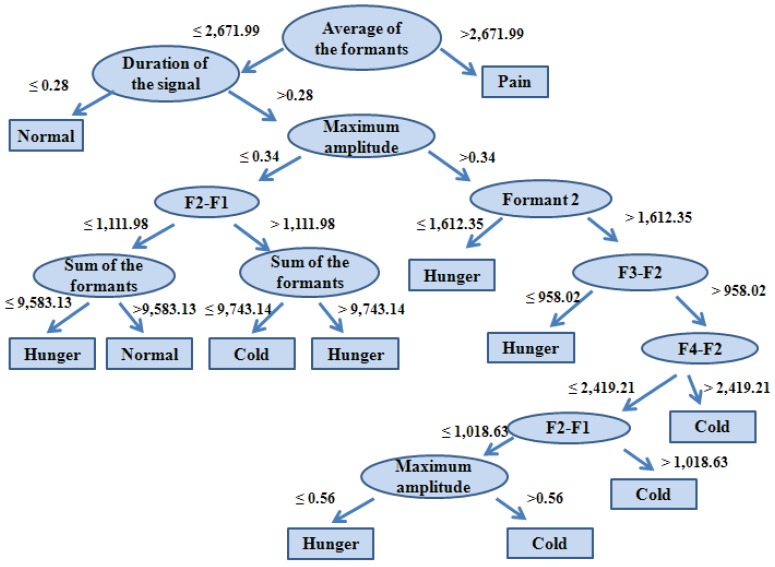
Decision tree generated by the C4.5 algorithm.

The decision tree was generated using the C4.5 algorithm, considering that the minimum number of objects per leaf is equal to nine (Figure 7). Through the generated rules, it was possible to classify the four types of distress with an accuracy rate of 81.69%. The precision rate (a type of accuracy for a specific class of the data) can be thought of as a measure of exactness. The class with the highest precision was the pain distress (0.99), followed by the normal welfare status (baseline) (0.90), the cold distress (0.89), and the hunger distress (0.69) (Table 3).

**Table 3:** Precision rates for the four classes studied.

Class	Precision
Normal (baseline)	0.82
Cold	0.71
Pain	0.98
Hunger	0.69

Twelve rules were generated from the decision tree (Table 4), and they may aid the development of an algorithm for the automatic identification of distress in piglets. From these rules, we enhance rules number 1, 3 and 12 which were sufficient for identifying the animal in pain.

**Table 4:** Description of the rules found within the decision tree.

Rule	Description
1	If the average value of the formants is ≤2,671.99 and the duration of the signal is ≤0.28, then the piglet is in normal condition.
2	If the average of formants is ≤2,671.99 and the duration of the signal is >0.28 and the maximum amplitude is ≤0.34 and F2- F3 is ≤1,111.98 and the sum of the formants is ≤9,583.13 then the piglet is hungry.
3	If the average of formants is ≤2,671.99 and the duration of the signal is >0.28 and the maximum amplitude is ≤0.34 and F2-F3 is ≤1,111.98 and the sum of the formants is >9,583.13 then the piglet is in normal condition.
4	If the average of formants is ≤2,671.99 and the duration of the signal is >0.28 and the maximum amplitude is ≤0.34 and F2-F3 is >1,111.98 and the sum of the formants is ≤9,743.14 then the piglet is feeling cold.
5	If the average of formants is ≤2,671.99 and the duration of the signal is >0.28 and the maximum amplitude is ≤0.34 and F2-F3 is >1,111.98 and the sum of the formants is >9,743.14 then the piglet is hungry.
6	If the average of formants is ≤2,671.99 and the duration of the signal is >0.28 and the maximum amplitude is >0.34 and the formant 2 is ≤1,612.35 then the piglet is hungry.
7	If the average of formants is ≤2,671.99 and the duration of the signal is >0.28 and the maximum amplitude is >0.34 and the formant 2 is >1,612.35 and F3-F2 is ≤958.02 then the piglet is hungry.
8	If the average of formants is ≤2,671.99 and the duration of the signal is >0.28 and the maximum amplitude is >0.34 and the formant 2 is >1,612.35 and F3-F2 is >958.02 and F4-F2 is ≤2,419.21 and F2-F1 is ≤1,018.63 and the maximum amplitude ≤0.56 then the piglet is hungry.
9	If the average of formants is ≤2,671.99 and the duration of the signal is >0.28 and the maximum amplitude is >0.34 and the formant 2 is >1,612.35 and F3-F2 is >958.02 and F4-F2 is ≤2,419.21 and F2-F1 is ≤1,018.63 and the maximum amplitude is >0.56 then the piglet is feeling cold.
10	If the average of formants is ≤2,671.99 and the duration of the signal is >0.28 and the maximum amplitude is >0.34 and the formant 2 is >1,612.35 and F3-F2 is >958.02 and F4-F2 is ≤2,419.21 and F2-F1 is >1,018.63 then the piglet is feeling cold.
11	If the average of formants is ≤2,671.99 and the duration of the signal is >0.28 and the maximum amplitude is >0.34 and the formant 2 is >1,612.35 and F3-F2 is >958.02 and F4-F2 is >2,419.21 then the piglet is feeling cold.
12	If the average of formant is >2,671.99 then the piglet is in pain.

Piglets respond to pain distress with loud squeals [2,18]. This type of call can be identified using only one attribute, which is the average frequency of formants. According to the generated tree, if the average of the formants frequencies is greater than 2,671.99, then the animal is in pain, and if this attribute is lower, then the animal is pain-free. Evaluating the frequencies of the first four formants of vocalizations from healthy and arthritic pigs, it was found that the frequencies from the first two harmonics, belonging to the healthy animals were larger than in the arthritic ones [19]. However, the frequencies of the third and fourth formants from healthy animals were smaller than in the arthritic ones. The C4.5 algorithm used the formants averages instead of separated frequencies, which showed a strong relationship with the pain situation. Excited pigs produce high tone vocalizations [7], and piglets in pain distress can be differentiated by the altered types of calls such as squeals that significantly differ from the other calls [2]. Furthermore, animals in pain distress also emit longer vocalizations [19] and could be distinguished from the others, even by the human ear. Nevertheless, it was not possible to confirm differences among the other tested distresses just by hearing the animals. The cold and hunger distresses were the most difficult to classify; it was necessary to use more attributes and combinations between them (Figure 7). 

The parameters of energy emission, frequency and duration of calls are particularly suitable for characterizing the type of call [2]. In the present study, the chosen algorithm did not use the energy parameter, but used the maximum range, which is related to the energy of the signal. Previously, variations have been found in the ranges of vocalizations from pigs in alert status (50–72 dB), apprehension (63–70 dB), and holding (74–120 dB) conditions [3]. The frequencies of the formants have been widely used by the algorithm, which is in line with [20], which also found that the differences in the resonance frequencies of pigs’ vocalization rely on the aversive stimulus. It was previously reported [21] that it is possible to recognize physiological changes by analyzing the characteristics of the vocal signals frequencies. 

Other studies [22] suggested algorithms using a combination of sound analysis by linear prediction coding and artificial neural networks to detect the animals’ stress vocalizations in commercial piggeries. In this research we used the process of data mining to develop an algorithm using animal vocalization to classify stress situations. These results could have been validated by physiological parameters. Thus, seeking to replace the physiological indicators by vocalization characteristics, as the techniques to obtain the physiological responses can often cause stress to the animals. Traits such as behavior, performance and physiological parameters (both rectal and surface temperatures, and respiratory rate) as indicators of the welfare status of pigs reared on a concrete floor bedded with coffee husks have been found in the current literature [23]. Differences in cortisol levels between the pigs submitted to the pre-slaughter and stunning stress and those ones that were not exposed to this condition was reported, suggesting that cortisol level might be also used as welfare status indicator [24]. Other authors [25] evaluated the Phosphocreatine Kinase (PCK), the Lactate Dehydrogenase enzymes and the glucose in blood samples, in addition to the salivary cortisol levels, normal heart rate, and heart rate during the pre-slaughter handling of pigs, and found that the pre-slaughter resting period significantly influenced the concentrations of lactate in the blood and the salivary cortisol levels. However, during the transportation, the heart rate was greater than elsewhere evaluated. Future studies should compare the vocalization data with the physiological parameters seeking a more reliable system for assessing the welfare status of pigs in commercial farming. 

## 4. Conclusions

The vocalization of piglets is an efficient technique for identifying their stress conditions. Specific computational techniques, such as data mining are needed for interpretation of animals’ vocalization. Determining the signals’ duration, the resonance frequencies, and signals’ amplitude in this research was fundamental for the interpretation of the sounds emitted by the piglets. The classification task can potentially aid in the discovery of relationships between the characteristics of acoustic signals and distress. 

## References

[1] Weary D.M., Fraser D. (1995). Signaling need: Costly signals and animal welfare assessment. Appl. Anim. Behav. Sci..

[2] Marx G., Horn T., Thielebein J., Knubel B., Borell E. (2003). Analysis of pain-related vocalization in young pigs. J. Sound Vib..

[3] Moura D.J., Silva W.T., Nääs I.A., Tolon Y.B., Lima K.A.O., Vale M.M. (2008). Real time computer stress monitoring of piglets using vocalization analysis. Comput. Electron. Agric..

[4] Schön P.C., Puppe B., Manteuffel G. (2004). Automated recording of stress vocalisations as a tool to document impaired welfare in pigs. Anim. Welf..

[5] Manteuffel G., Puppe B., Schön P.C. (2004). Vocalization of farm animals as a measure of welfare. Appl. Anim. Behav. Sci..

[6] Cordeiro A.F.S., Pereira E.M., Nääs I.A., Silva W.T., Moura D.J. (2009). Medida de vocalização de suínos (Sus scrofa) como um indicador de gasto energético. Braz. J. Biosyst. Eng..

[7] Schrader L., Todt D. (1998). Vocal quality is correlated with levels of stress hormones in domestic pigs. Etology.

[8] Johnson R.W., von Borell E., Anderson L.L., Kojic L.D., Cunnick J.E. (1994). Intra cerebro ventricular injection of corticotrophin-releasing hormone in the pig: Acute effects on behavior, adrenocorticotropin secretion, and immunosuppression. Endocrinology.

[9] Kranendonk G., Hopster H., Fillerup M., Ekkel E.D., Mulder E.D. (2006). Cortisol administration to pregnant sows affects novelty-induced locomotion, aggressive behaviour, and blunts gender differences in their offspring. Hormone. Behav..

[10] Nääs I.A., Campos L.S.L., Baracho M.S., Tolon Y.B. (2008). Uso de redes neurais artificiais na identificação de vocalização de suínos. Engenharia Agrícola.

[11] Lima M.G.F., Rodrigues L.H.A. (2010). Árvore de decisão aplicada em dados de incubação de matrizes de postura Hy-Line w36. Ciência e Agrotecnologia.

[12] Pandorfi H., Silva I.J.O., Sarnighausen V.C.R., Vieira F.M.C., Nascimento S.T., Guiselini C. (2011). Uso de redes neurais artificiais para predição de índices zootécnicos nas fases de gestação e maternidade na suinocultura. Revista Brasileira de Zootecnia.

[13] Fayyad U., Piatetsky-Shapiro G., Smyth P. (1996). From data mining to knowledge discovery: An overview. Artif. Intell. Mag..

[14] Curtis S.E. (1974). Responses of the Piglet to Perinatal Stressors. J. Anim. Sci..

[15] Hall M., Frank E., Holmes G., Pfahringer B., Reutemann P., Witten I.H. (2009). The WEKA Data Mining Software: An Update. SIGKDD Explorations. http://www.cs.waikato.ac.nz.

[16] Han J., Kamber M., Pei J. (2011). Data Mining: Concepts and Techniques.

[17] Landis J.R., Koch G.G. (1977). The measurement of observer agreement for categorical data. Biometrics.

[18] Leidig M.S., Hertrampf B., Failing K., Schumann A., Reiner G. (2009). Pain and discomfort in male piglets during surgical castration with and without local anesthesia as determined by vocalization and defence behaviour. Appl. Anim. Behav. Sci..

[19] Risi N. (2010). Uso da vocalização como indicador patológico em leitões na fase de maternidade. M.S. Thesis.

[20] Dupjan S., Schön P.C., Puppe B., Tuchscherer A., Manteuffel G. (2008). Differential vocal responses to physical and mental stressors in domestic pigs (*Sus scrofa*). Appl. Anim. Behav. Sci..

[21] Ikeda Y., Ishii Y. (2008). Recognition of two psychological conditions of a single cow by her voice. Comput. Electron. Agric..

[22] Manteuffel G., Schön P.C. (2004). STREMODO, an innovative technique for continuous stress assessment of pigs in housing and transport. Archiv Tierzucht.

[23] Caldara F.R., Rosa P.S.G., Ferreira R.A., Reis N.M.O., Nääs I.A., Paz I.C.L.A., Garcia R.G., Ferreira V.M.O.S. (2012). Behavior, performance and physiological parameters of pigs reared in deep bedding. Engenharia Agrícola.

[24] Santana A.P., Murata L.S., Mc Manus C.P., Bernal F.E.M. (2009). Dosagem de cortisol sanguíneo em suínos submetidos ao manejo pré-abate e insensibilização elétrica. Arch. Zootecnia.

[25] Dalla Costa O.A., Ludke J.V., Coldebella A., Kich J.D., Costa J.R.P., Faucitano L., Peloso J.V., Roza D.D. (2009). Efeito do manejo pré-abate sobre alguns parâmetros fisiológicos em fêmeas suínas pesadas. Ciência Rural.

